# Peer Review of "Cognitive Factors Associated With Public Acceptance of COVID-19 Nonpharmaceutical Prevention Measures: Cross-sectional Study"

**DOI:** 10.2196/37121

**Published:** 2022-05-13

**Authors:** Mathew Mbwogge

**Keywords:** Extended Parallel Process Model, COVID-19, lockdown, public acceptance, nonpharmaceutical measures, Likert scale, France

*This is a peer-review report submitted for the paper “Cognitive Factors Associated With Public Acceptance of COVID-19 Nonpharmaceutical Prevention Measures: Cross-sectional Study.”*

## Round 1 Review

### General Comments

The COVID-19 pandemic has led to huge psychological and social repercussions [1-3], affecting both the way people interact and their perception of the pandemic [4]. Nonpharmaceutical interventions including social distancing, stay-at-home orders, and curfews were found to be effective measures for reducing the number of cases [5] but may have increased psychological [6] and emotional [7] distress. The evaluation of nonpharmaceutical COVID-19 measures through the involvement of the public is key to determining their effectiveness and impact, as this may help develop more effective and user-friendly interventions.

The authors of the paper “Acceptance of COVID-19 preventive measures as a trade-off between health and social outcomes” [8] investigated the acceptance of COVID-19 preventive measures and its association with COVID-19 perception among 2004 subjects and found the acceptance rates for personal protective measures and collective measures to be 86.1% and 70%, respectively. They also found that acceptance of measures was positively associated with perceived efficacy, perceived severity, and fear. Other studies that investigated the public’s acceptance of preventive measures found that moral considerations predicted higher acceptance for collective measures compared to personal considerations [9] and that men and younger individuals showed lower acceptance of preventive measures [10]. In addition, trust in science was found to be a greater predictor of adoption than trust in politics [10].

The paucity of published literature regarding this subject makes the present paper of high interest to the journal’s readership. The paper’s overall structure is in accordance with the journal’s IMRD structure. The Abstract is well structured, summarizing the main points of the paper. The introduction is well-articulated in relation to implemented measures (with dates) and their evolution and is supported with references. The reported methods seem convincing, making use of the renowned Likert scale [11] in measuring the public’s agreement to measures, as well as the Extended Parallel Process Model [12] that has also been deployed in other studies that examined the impact of preventive interventions [13]. There is a good flow in the data analysis justified with references, with an explanation of how they moved from statistically significant variables in model 1 to loading the multivariate model and, last but not least, the authors made the data readily available, which altogether gives meaning to the presented results. The discussion is well structured, even though informal, starting with the key findings followed by the interpretation, limitations, and conclusion. The English used is simple enough for the readership’s understanding of the paper.

That said, this paper needs to be improved to better align with the journal’s guidelines and be more appealing to its readership. Kindly refer to the specific comments below.

### Specific Comments

1. Your title needs to follow the guidelines of the journal to which you are submitting.

2. The “Background” and “Methods” subsections of your Abstract need to be improved.

3. The specific objectives of the paper need to stand out as a subsection.

4. Major subsections are missing in your introduction, methods, and the results.

5. Some subsections in the Methods section warrant improvement.

6. The structure of the Discussion section needs to align with the guidelines.

7. The in-text citations and references must comply with the journal’s guidelines.

8. Tables and figures in the appendix need to be moved to the body of the text.

#### Major Comments

1. Format your title to include the country and study design. Kindly refer to the guidelines for titles [14]. For instance, “Acceptance of COVID-19 preventive measures as a trade-off between health and social outcomes in France: Cross-sectional Study”. By the way, I have not seen anywhere in the body of your paper where health and social outcomes mentioned in your title have been articulated.

2. The beginning of your background in the Abstract (“A better understanding of the factors underlying their acceptance may contribute greatly to the design of more effective public health programs during the current and future pandemics”) does not make it clear to the reader to whom you are alluding. Kindly rephrase.

3. Your objectives need to be improved. I guess along the lines of (1) measure the public’s acceptance of COVID-19 preventive measures and (2) assess the association of the public’s acceptance of these measures and their perception of COVID-19.

4. In the “Methods” subsection of your Abstract, kindly add a summary of how data for each objective was analyzed and the statistical package that was used to perform the analysis. Please note that your Abstract (currently

5. It would be good to include the following items under Introduction after the background: (1) study rationale, to justify your study and to present the Extended Parallel Process Model, and (2) specific objectives, to clearly outline your study objectives.

6. Kindly start your Methods section with a subsection “Study Design” and specify your study design.

7. The statement under Participants and Procedures—that is, “The objective of the research was to assess the emotional, cognitive, and behavioral responses of the French people to the COVID-19 epidemic during the full lockdown (wave 1) and thereafter (wave 2)”—should not be there. You might want to move this to the study aim or specific objectives.

8. The second to last statement under Participants and Procedures (“For this study, we analyzed data from a 2-week survey administered 6-8 weeks after the first lockdown between June 25 and July 5, 2020”) does not fit quite well under this subsection. I suggest you rephrase as “This was a 2-week survey administered 6-8 weeks after the first lockdown of June 25 through July 5, 2020” and incorporate it into your Study Design subsection.

9. The last sentence under Participants and Procedures needs to be moved to a section entitled “Ethical Considerations” to be created at the end of the Methods section (just before the Results section).

10. Kindly start your Results section with the subsection “Participant Characteristics” to give a summary of participant characteristics. Kindly move your Table 1 in the appendix to accompany your participant characteristics.

11. You need to move Tables 2-4 in the appendix to where they are first mentioned in the Results section for easy comprehension. It becomes easy to refer to the tables while reading. In addition, bear in mind that you are allowed to include up to a total of 5 tables in the body of your text.

12. Move Figure 1 to where it is first mentioned in your Results section.

13. Kindly organize your Discussion into (1) Principal Results, (2) Comparison With Prior Studies, (3) Study Limitations, and (4) Conclusion.

14. The in-text citations and references must be in line with the AMA citation style, in accordance with the journal guidelines [15]. Kindly refer to the references accompanying this report.

#### Minor Comments

15. Based on your title, I guess your study aimed to evaluate the acceptance of COVID-19 nonpharmaceutical measures. I suggest you add to your background (both in the Abstract and the Introduction) a study aim similar to the above and use the last sentence of your background in the Abstract to create a separate “Objectives” subsection before the Abstract’s “Methods” subsection.

16. I suggest you rephrase sentence #2 in the methods subsection of your Abstract as “For objective 1, participants were asked the extent to which they supported 8 COVID-19 preventive measures using a 4-point Likert scale”, and start the following sentence with “For objective 2, COVID-19 perceptions…”

17. In the results subsection of the Abstract, could you please include figures for positive and negative associations and highlight if these were statistically significant or not?

18. Kindly include “Likert scale”, “France” and “Nonpharmaceutical measures” in your keywords.

19. Under Measurements, kindly substantiate your use of the Likert scale with suitable references. You might want to use this link [16].

20. For your beginning statement under Data Analysis, I suggest you use “frequencies (N)” instead of “numbers (N)”.

21. I like the flow and harmony between Participants and Procedure, Measurements, and Data Analysis. You did well to have organized these by objective. In your Data Analysis, could you please highlight how you assessed the model fit (goodness of fit) of your multivariate model?

22. I suggest you organize your Results section, which already is in good shape, by study objective after “Participant Characteristics” so that it flows well in the measurements and data analysis subsections.

23. Relating your study results to the title, readers might expect to see where you articulated the trade-off between health and social outcomes. This is not the case. It might be worthwhile to rephrase your title.

24. Kindly format your tables [17] and figures [18] following the journal guidelines.

25. I suggest you start your Conclusion by highlighting the study objectives.

26. It is important to include citations from the journal to which you are submitting or its sister journals.

## Round 2 Review

### General Comments

The authors of the paper titled “Cognitive Factors Associated With Public Acceptance of COVID-19 Nonpharmaceutical Prevention Measures: Cross-sectional Study” [8] have implemented the recommendations to the letter. However, a new and close look warrants a few more modifications. Kindly refer to minor comments below.

### Specific Comments

#### Major Comments

1. The phrase “The aim of this study was to evaluate the acceptance of COVID-19 nonpharmaceutical prevention measures in France”, in the Objectives subsection should be moved to be the last sentence of the Background subsection in your Abstract.

2. Under Rationale, I think you should start the second sentence as “This study was based on the Extended Parallel Process Model.”

3. The last sentence of your Rationale is not suitable for this section, so I suggest removing it.

4. The starting sentence of your Specific Objectives should be part of your Rationale instead, so you may want to move that from there.

5. All weblinks in the body of your text should be cited as references. The journal to which this manuscript is submitted does not allow the use of weblinks in the body of the text.

6. The phrases “EPPM factors were estimated using an unweighted least-square factorial analysis, followed by a Promax rotation, and 5 factors were extracted accordingly” and “The raw scale scores were transformed to a 0-100 scale. Higher scores in the respective scales are indicative of greater perceived efficacy, lack of fear control, severity, susceptibility, or avoidance” should be moved to Data Analysis.

7. Tables 1, 3, and 4 still need to be updated to comply with the journal guidelines. You will notice in this link [17] that item categories like “Age in years” and “Professional status” should be in their own row while the items under each category start on the next row.

8. As part of the participant characteristics, kindly include the mean age of participants and if the mean age difference between men and women was statistically significant.

9. Regarding your statement “The raw scale scores were transformed to a 0-100 scale”, there is a serious debate about calculating Likert scale scores from responses. Kindly be clear on how you converted the responses to scores.

10. Kindly include your Figure 1 in the body of the text. All figures uploaded online must also be included in the body of the text, as per the guidelines.

11. Kindly move the first sentence of your Principal Results (“The aim of this study was to evaluate the acceptance of COVID-19 nonpharmaceutical measures and, more specifically, to measure the public’s acceptance of these measures and their association with COVID-19 perceptions”) to be the starting sentence of your Conclusion.

12. Kindly ensure that all percentages reported in the body of your text (apart from those from other studies) are expressed in absolute values in parentheses; for instance, 20% (5/25).

13. Evidence suggests that there are also issues around sex and gender reporting [19-21]. Since sex is biological, it will be good to make clear in your methods that the sex definition was based on self-reported sex [20].
